# Predicting Simultaneous Heart Kidney Allocation and Posttransplant Adverse Kidney Outcomes

**DOI:** 10.1016/j.ekir.2025.10.005

**Published:** 2025-10-15

**Authors:** Mutlu Mete, Mehmet U.S. Ayvaci, Ahmet B. Gungor, Faris Araj, Deepak Acharya, Benjamin Hippen, Xingxing S. Cheng, Miklos Z. Molnar, Tarek Alhamad, Enver Akalin, Neeraj Singh, Prince M. Anand, Gaurav Gupta, Matthias Peltz, Venkatesh K. Ariyamuthu, Abd A. Qannus, Iyad S. Mansour, Maryam Emami, Vikas Pal, Bekir Tanriover

**Affiliations:** 1 Department of Information Science, University of North Texas, Denton, Texas, USA; 2 Information Systems, Naveen Jindal School of Management, The University of Texas at Dallas, Richardson, Texas, USA; 3 Division of Nephrology, Department of Medicine, The University of Arizona, Tucson, Arizona, USA; 4 Division of Cardiology, Department of Medicine, University of Texas Southwestern Medical Center, Dallas, Texas, USA; 5 Division of Cardiology, Department of Medicine, The University of Arizona, Tucson, Arizona, USA; 6 Transplant Medicine, Global Medical Office, Fresenius Medical Care, Charlotte, North Carolina, USA; 7 Division of Nephrology, Department of Medicine, Stanford University, Stanford, California, USA; 8 Division of Nephrology and Hypertension, Department of Medicine, Spencer Fox Eccles School of Medicine at the University of Utah, Salt Lake City, Utah, USA; 9 Division of Nephrology, Department of Medicine, Washington University in St. Louis, St. Louis, Missouri, USA; 10 Division of Nephrology, Department of Medicine, Montefiore Medical Center, Albert Einstein College of Medicine, Bronx, New York, USA; 11 Division of Nephrology, Department of Medicine, Louisiana State University, Shreveport, Louisiana, USA; 12 Division of Nephrology, Department of Medicine, Medical University of South Carolina, Lancaster, South Carolina, USA; 13 Division of Nephrology, Department of Medicine, Virginia Commonwealth University, Richmond, Virginia, USA; 14 Department of Cardiovascular and Thoracic Surgery, University of Texas Southwestern Medical Center, Dallas, Texas, USA; 15 Biostatistics, Department of Medicine, The University of Arizona, Tucson, Arizona, USA

**Keywords:** allocation, heart transplant, machine learning, prediction, random forest, simultaneous heart kidney transplantation

## Abstract

**Introduction:**

For individuals with both end-stage heart failure and end-stage kidney disease or persistent acute kidney injury (AKI), simultaneous heart-kidney transplantation (SHKT) emerges as a viable treatment option, potentially yielding superior survival rates compared with heart transplantation (HT) alone. Nevertheless, accurately forecasting kidney recovery following HT in patients with moderate kidney failure poses challenges, thereby complicating the decision-making process for SHKT.

**Methods:**

This study employed a random forest (RF) machine learning algorithm, using 15 variables with the highest feature importance scores in the Organ Procurement and Transplantation Network (OPTN) data in which we analyzed a retrospective cohort of adult HT recipients from October 18, 2018 to December 31, 2020 in the US, with a follow-up for at least 1 year. The algorithm’s goal was to predict a composite binary outcome with a calculated probability. An adverse outcome included the need for SHKT or adverse kidney outcomes within the first-year posttransplant (defined as end-stage kidney disease requiring chronic dialysis, glomerular filtration rate (GFR) ≤ 20 ml/min per 1.73 m^2^ or listing for retransplant). The model underwent both internal and external validation.

**Results:**

Of the 6579 patients in the study cohort, 13.4% received SHKT or experienced adverse kidney outcomes within a year following HT (*n* = 880). The RF model demonstrated a high specificity (0.941–0.955) and negative predictive value (0.940–0.955). However, it exhibited a moderate level of sensitivity (0.605–0.694) and positive predictive value (0.604–0.680). The concordance (c)-statistics ranged between 0.849 and 0.899, indicating effective class differentiation.

**Conclusion:**

This tool supplements, not replace, clinical judgment in addressing the complexities of SHKT decision-making at the time of waitlisting.

## Graphical abstract

**Figure:**
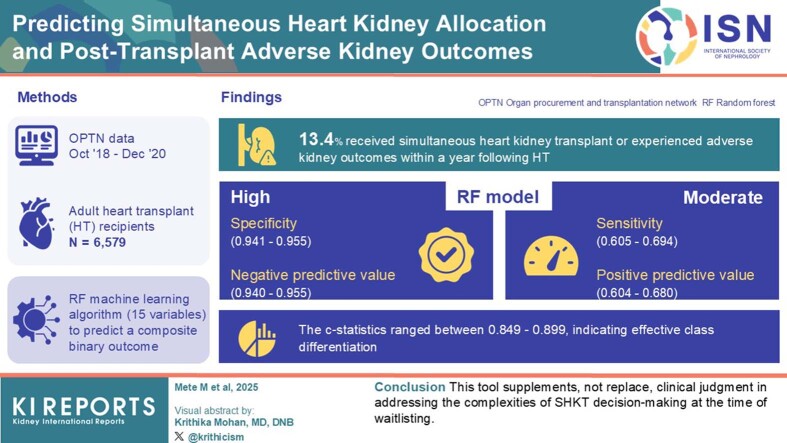

Patients undergoing HTs often experience AKI leading to chronic kidney disease (CKD) which is often associated with higher mortality rates and increased health care costs.^1^ For those with end-stage heart failure and end-stage kidney disease or persistent AKI, SHKT presents an effective treatment option and offers better survival outcomes compared to HT alone.^2^, ^3^, ^4^, ^5^

SHKT candidates undergo evaluation by transplant nephrologists and their assessments involve detailed query of kidney function trend (serial serum creatinine and cystatin-c estimated GFR (eGFR) measurements, 24-hour urine creatinine clearance, and rarely nuclear medicine scans and iohexol/iothalamate plasma/urine clearance), proteinuria quantification, kidney imaging (kidney length and cortex size, and volume measurements), kidney biopsy findings, and duration of renal replacement therapy, if on dialysis.^6^^,^^7^ Currently, most patients with end-stage heart failure on hemodialysis at the time of transplant undergo SHKT (currently accounting for approximately 40% of SHKTs).^8^^,^^9^ However, there is no evidence-based consensus on the best transplant strategy for patients with moderate to severe CKD and/or persistent AKI on the waitlist, because predicting kidney recovery post-HT is challenging and decision making for SHKT is complicated when randomized controlled trials are lacking.^6^^,^^10^, ^11^, ^12^

The landscape of SHKT has been significantly shaped by 2 key developments in the past 5 years. The first is the implementation of the new heart allocation policy on October 18, 2018, which led to a substantial increase in SHKTs, accounting for approximately 9% of all HTs performed in the US between 2021 and 2023 (Figure 1).^9^^,^^13^ This policy, aimed at reducing mortality among critically ill patients with heart failure, many of whom also suffer from acute and chronic kidney dysfunction, has encouraged wider organ sharing. However, it has also raised concerns reducing posttransplant survival without providing improvement in waitlist mortality for SHKT recipients.^8^^,^^9^^,^^14^, ^15^, ^16^ The second major development is the adoption of Medical Eligibility and Safety Net criteria for SHKT and simultaneous lung-kidney transplant by the OPTN on September 28, 2023. These criteria, modeled after those for simultaneous liver-kidney transplants established in 2017, aim to standardize multiorgan allocation.^17^^,^^18^ They set out specific eligibility conditions for heart and lung transplant candidates based on retrospective studies.^2^^,^^3^^,^^5^^,^^6^ However, these criteria fall short in providing recommendations backed by data from prospective evidence-based studies.^8^^,^^19^^,^^20^

**Figure 1:**
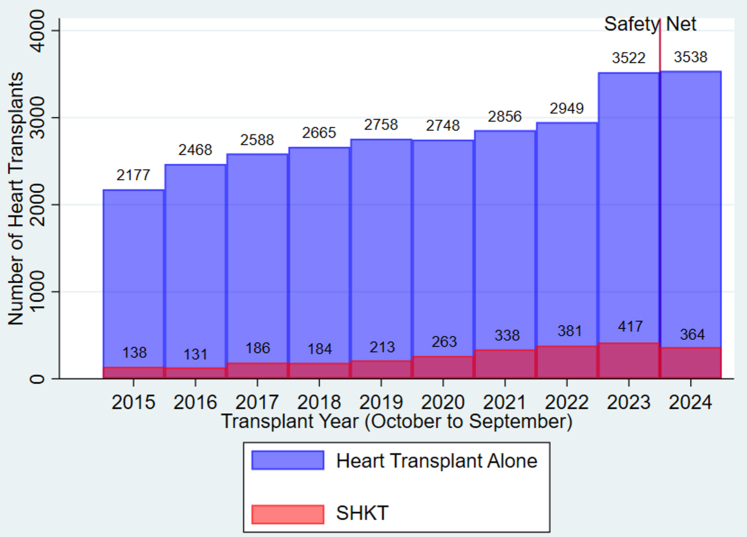
Trends in heart-alone and simultaneous heart-kidney transplants in the United States, 2015–2024. SHKT, simultaneous heart-kidney transplants.

Although the comprehensive assessment by a transplant nephrologist to evaluate renal reserves and the reversibility of AKI remains crucial in the allocation of SHKT, the integration of an artificial intelligence (AI)-based predictive algorithm may assist transplant surgeons and physicians at the time of waitlisting to streamline the decision-making process and reduce uncertainty when choosing between SHKT and HT alone, given the severe shortage of kidneys.^21^^,^^22^ Thus, we have developed a machine-learning algorithm, using an RF model, designed to support clinical decisions at the time of heart transplant listing. Its primary function is to predict a binary outcome with probability that includes the need for SHKT and the likelihood of adverse kidney outcomes within 1 year posttransplant. To facilitate accessibility for transplant surgeons and physicians, we have implemented this algorithm into a user-friendly, web-based decision tool at the patient level.

## Methods

This study received approval from the Institutional Review Board at the University of Arizona, which waived the requirement for informed consent because of the study's retrospective and limited data analysis nature. No animal data was included. We adhered to the STROBE and STARD guidelines for reporting observational epidemiologic studies and diagnostic accuracy, respectively, and complied with the World Medical Association's Statement on Organ and Tissue Donation, the Declaration of Helsinki, and the Declaration of Istanbul on Organ Trafficking and Transplant Tourism.

The data that support the findings of this study are available from the corresponding author upon reasonable request.

### Data Source

This study used data from the United Network for Organ Sharing Standard Transplant Analysis and Research files, which encompass information on all donors, candidates on the waiting list, and transplant recipients in the USA. The OPTN operates under the oversight of the Health Resources and Services Administration of the US Department of Health and Human Services.

### Study Participants

Our algorithm was developed using a retrospective cohort of adult heart transplant recipients including SHKTs from the OPTN national registry. This cohort comprised 7878 patients between October 18, 2018, and December 31, 2020. For external validation, we used data from HTs performed between January 1 and June 30, 2021 (*n* = 1567), and from HTs performed between September 28, 2023, and March 30, 2024 (postpolicy cohort, specifically the Medical Eligibility and Safety Net policy) (*n* = 1853). All patients were followed-up with for a minimum of 1 year, and an additional 6-month administrative censoring period was implemented to reduce ascertainment bias.^23^ The study excluded pediatric recipients (aged < 18 years), patients with previous nonheart transplants, and the ones with missing creatinine values and/or dialysis status at the time of listing or transplant for both cohorts. The final study cohort included 6579 patients (October 18, 2018–December 31, 2021), along with 2 additional external validation cohorts comprising 1567 and 1853 patients, respectively.

### Primary Outcome

We aimed to predict a composite binary outcome with an associated risk score (a probability) at the time of heart transplant listing for all heart transplant candidates. The adverse kidney outcome comprises HT patients who underwent SHKTs or experienced adverse kidney outcomes within 1-year post-HT, representing those who could have been allocated a kidney. Adverse kidney outcomes are defined as end-stage kidney disease requiring chronic dialysis, an eGFR < 20 ml/min per 1.73 m^2^ or needing a kidney transplant within the first year of HT alone transplantation. HT-alone transplants without adverse kidney outcomes 1-year posttransplant are classified as nonadverse outcomes. The model is intended to support clinical decision-making at the time of listing, before determining between HT alone and SHKT.

### Test Methods

**Figure 2:**
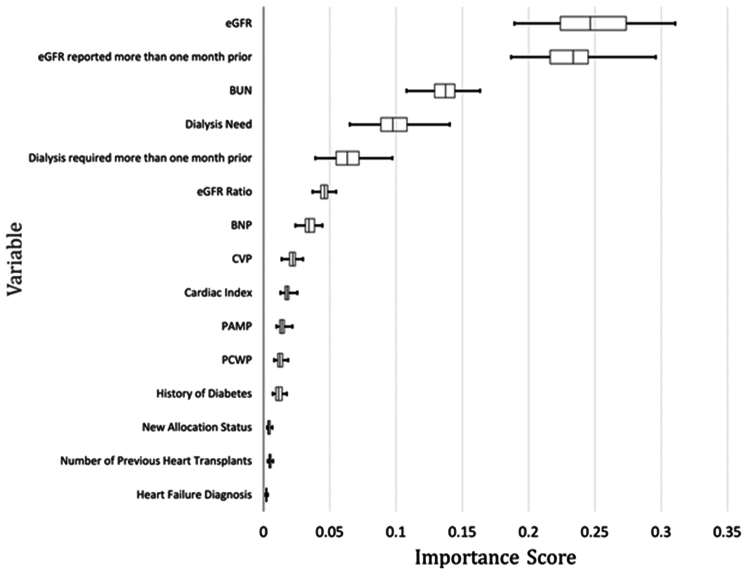
Box plot of the variable contribution in the model using 1000 runs of random forest algorithm parameters (including number of trees, max depth, max features, class weight, random seed). BNP, B-type natriuretic peptide; BUN, blood urea nitrogen; CVP, central venous pressure; eGFR, estimated glomerular filtration rate; PAMP, pulmonary artery mean pressure; PCWP, pulmonary capillary wedge pressure.

We designed an RF machine learning algorithm^24^ using an open-source software kit (Scikit-learn random forest package) for our prediction task. This advanced ensemble algorithm amalgamates predictions from numerous decision trees it generates. Initially, an expert panel (transplant surgeon, cardiologist, and nephrologist) selected 44 key variables from 531 available in the United Network for Organ Sharing Standard Transplant Analysis and Research waitlist and transplant files (Supplementary Table S1), deemed essential for SHKT decision-making. Subsequently, the algorithm computed the “feature importance” of these variables in the training phase using the Gini importance method. This led us to develop a more streamlined algorithm, incorporating the top 15 variables (Figure 2). All variables were extracted from the United Network for Organ Sharing Standard Transplant Analysis and Research file at the time of listing unless stated otherwise. However, eGFR and dialysis status were recorded at 2 separate time points: at the time of waitlisting and before transplantation, approximately 35 to 40 days apart, representing the median waiting time for HT. The top 15 variables include eGFR, dialysis status, eGFR ratio (eGFR at listing / eGFR prior to transplant), blood urea nitrogen, B-type natriuretic peptide (BNP) or N terminal pro-BNP, right heart catheter values (central venous pressure, pulmonary mean arterial pressure, pulmonary capillary wedge pressure, cardiac index), history of diabetes, the new heart transplant allocation status (6-tier), previous heart transplant, and heart failure diagnosis category. eGFRs were calculated using the race-free 2021 CKD-EPI creatinine equation.^25^ The RF model assumes an eGFR of 10 ml/min per 1.73 m^2^ for patients on dialysis, based on the premise that patients with an eGFR at or below this level will benefit from renal replacement therapy. The median time for the reported laboratory values and right heart catheterization measurements was the same day for renal function testing (blood urea nitrogen and eGFR) and within 5 days of each other for the BNP test.

The final model predicts the binary outcome as either an adverse and nonadverse and calculates an associated risk score, expressed as a probability between 0 and 1. A probability of 0.00 to 0.49 indicates a nonadverse outcome, 0.50 signifies equipoise, and 0.51 to 1.00 represents an adverse outcome. To convert the risk score to a percentile, we multiplied the probability by 100 for adverse outcomes and (1 – probability) by 100 for nonadverse outcomes to keep them in the scale (range: 50% – 100%). The interpretation of the risk score based on the RF model's prediction is as follows: a score of 0 corresponds to a web-calculator display of nonadverse 100%, a score of 0.1 corresponds to nonadverse 90%, and a score of 0.4 corresponds to nonadverse 60%. A score of 0.5 indicates equipoise 50%, whereas a score of 0.8 corresponds to adverse 80%.

We used the K-nearest neighbors’ algorithm^26^ (K = 50) from the Scikit-Learn library to impute missing values (Scikit-Learn library, the sklearn.impute.KNNImputer module),^27^ primarily applied to right heart catheterization measurements (9.8%), BNP (13.4%), and blood urea nitrogen (0.8%).

### Performance Assessment and Validation

We limited our search to models, achieving a minimum of 70% in both sensitivity and specificity. This exhaustive search, conducted on high-performance computing clusters, focused on selecting models with the highest area under the curve performance. For internal validation, we employed 5-fold cross-validation.^28^ We reported the model's sensitivity, specificity, negative predictive values (NPV), and positive predictive values (PPV), area under the curve for receiver operating characteristics or c-statistic, the area under the curve for the precision/recall (P/R) curve, and the precision/recall F1 score (the harmonic mean of precision and recall) using predictions from the cross-validated test cohorts. We conducted a comprehensive evaluation of the model’s performance in the following 2 distinct cohorts: (i) an external validation cohort of US heart transplant patients from January to June 2021 (*n* = 1567) and (2) a second external validation cohort of postpolicy Medical Eligibility and Safety Net patients from September 2023 to March 2024 (N = 1853). Analyses were performed using Python 3.11.0 (Python Software Foundation) and Stata 18 (StataCorp, College Station, TX).

## Results

In Table 1 and Supplementary Tables S2 and S3, we present the characteristics of the 6579 patients in our final study cohort, categorized by the primary outcome and transplant type. Of the total, 5699 patients (86.6%), referred to as the nonadverse outcome cohort, underwent only HT and did not experience any adverse kidney outcomes within the first-year posttransplant. In contrast, 880 patients (13.4%), known as the adverse outcome cohort, either received SHKTs (*n* = 540) or developed adverse kidney outcomes within a year of heart transplant alone (*n* = 340). The median age of the candidates was 57 years, and the majority were male (72.5%). In terms of race, Whites formed the largest group (62%), followed by African Americans (24%) and Hispanics (9.2%). The group with adverse outcomes exhibited significantly higher rates of diabetes, heart retransplants, pretransplant dialysis, increased right heart pressures, and elevated BNP or N terminal pro-BNP levels, alongside lower eGFRs. Notably, the posttransplant survival rate for 1 year was lower in the SHKT and adverse kidney outcome group (83.3%) compared with those who underwent heart transplant alone without adverse outcomes (92.6%, *P*-value < 0.001).

**Table 1:** Characteristics the heart transplant candidates received transplant between October 18, 2018 and December 31, 2020 in the United States

Characteristics	Study cohort	Heart transplant alone without adverse renal outcome within 1-yr posttransplant	SHKT and/or adverse renal outcome within 1-yr posttransplant	*P*-value^a^
*n* (%)	6579 (100)	5699 (86.6)	880 (13.4) [SHKT, *n* = 540]	
Age, median (IQR) yrs	57 (46–63)	56 (45–63)	57 (49–64)	< 0.001
Sex(male), *n* (%)	4767 (72.5)	4145 (72.7)	622 (70.7)	0.21
Race, *n* (%)				< 0.001
White	4082 (62.0)	3653 (64.1)	429 (48.8)	
Black	1578 (24.0)	1258 (22.1)	320 (36.4)	
Hispanic	608 (9.2)	531 (9.3)	77 (8.8)	
Asian	235 (3.6)	196 (3.4)	39 (4.4)	
Other	76 (1.2)	61 (1.1)	15 (1.7)	
Recipient height (cm), mean (SD)	173.8 (10.1)	173.9 (10.1)	173.3 (10.3)	0.44
Recipient weight (kg), mean (SD)	84.2 (18.4)	84.2 (18.4)	83.9 (18.4)	0.70
Body surface area (m^2^), mean (SD)	2.0 (0.2)	2.0 (0.2)	2.0 (0.2)	0.44
Body mass index (kg/m^2^)	27.8 (5.0)	27.7 (5.0)	27.8 (5.1)	0.61
History of diabetes, *n* (%)				< 0.001
No	4703 (71.5)	4189 (73.5)	514 (58.4)	
Type I	64 (1.0)	49 (0.9)	15 (1.7)	
Type II	1812 (27.5)	1461 (25.6)	351 (39.9)	
Etiology of cardiomyopathy, *n* (%)				< 0.001
Nonischemic	3660 (55.6)	3214 (56.4)	446 (50.7)	
Ischemic	1783 (27.1)	1540 (27.0)	243 (27.6)	
Restrictive	281 (4.3)	233 (4.1)	48 (5.5)	
Hypertrophic	214 (3.3)	197 (3.5)	17 (1.9)	
Congenital	357 (5.4)	315 (5.5)	42 (4.8)	
Failed heart transplant	210 (3.2)	133 (2.3)	77 (8.8)	
Others	74 (1.1)	67 (1.2)	7 (0.8)	
Previous heart transplant, *n* (%)				< 0.001
0	6355 (96.6)	5552 (97.4)	803 (91.3)	
1	207 (3.2)	139 (2.4)	68 (7.7)	
2	17 (0.3)	8 (0.1)	9 (1.0)	
Cardiac index (l/min/m^2^), mean (SD)	2.2 (0.6)	2.2 (0.6)	2.3 (0.7)	< 0.001
Central venous pressure (mm Hg), mean (SD)	9.4 (5.6)	9.2 (5.4)	11.1 (6.2)	< 0.001
Pulmonary capillary wedge pressure (mm Hg), mean (SD)	18.0 (8.3)	17.8 (8.3)	19.4 (8.3)	< 0.001
Pulmonary artery mean pressure (mm Hg), mean (SD)	27.3 (9.2)	27.0 (9.2)	29.1 (8.9)	< 0.001
Mechanical ventilation requirement, *n* (%)	134 (2.0)	120 (2.1)	14 (1.6)	0.31
ECMO, *n* (%)	219 (3.3)	189 (3.3)	30 (3.4)	0.88
IABP, *n* (%)	1017 (15.5)	884 (15.5)	133 (15.1)	0.76
VAD, *n* (%)				< 0.001
None	4825 (73.3)	4155 (72.9)	670 (76.1)	
LVAD alone	1631 (24.8)	1447 (25.4)	184 (20.9)	
RVAD	12 (0.2)	8 (0.1)	4 (0.5)	
TAH	22 (0.3)	17 (0.3)	5 (0.6)	
BiVAD	89 (1.4)	72 (1.3)	17 (1.9)	
BNP				
BNP (pg/ml), mean (SD), *n* = 3200	1028.7 (1429.8)	961.6 (1242.3)	1485.3 (2286.3)	< 0.001
NT-proBNP (pg/ml), mean (SD), *n* = 2498	4572.4 (6308.9)	3960.9 (5159.8)	8704.2 (10467.0)	< 0.001
BUN (mg/dl) at listing, mean (SD)	25.0 (13.9)	23.0 (11.2)	38.0 (20.9)	< 0.001
eGFR ml/min per 1.73 m^2^ at listing	65.9 (26.6)	70.1 (24.1)	38.2 (25.3)	< 0.001
eGFR ml/min per 1.73 m^2^ prior to transplant	65.9 (28.2)	70.7 (25.4)	34.9 (25.8)	< 0.001
eGFR ratio (wait listing/prior to transplant)	1.0 (0.5)	1.1 (0.5)	1.0 (0.4)	< 0.001
Dialysis at listing, *n* (%)	212 (3.2)	22 (0.4)	190 (21.6)	< 0.001
Dialysis prior to transplant, *n* (%)	358 (5.5)	83 (1.5)	275 (31.4)	< 0.001
eGFR ml/min per 1.73 m^2^ prior to transplant, if not on dialysis, median (IQR)	66.3 (50.1–87.2)	68.7 (52.9–88.9)	41.4 (28.2–60.1)	< 0.001
UNOS region				< 0.001
1	380 (5.8)	328 (5.8)	52 (5.9)	
2	623 (9.5)	562 (9.9)	61 (6.9)	
3	752 (11.4)	663 (11.6)	89 (10.1)	
4	576 (8.8)	490 (8.6)	86 (9.8)	
5	1077 (16.4)	901 (15.8)	176 (20.0)	
6	183 (2.8)	171 (3.0)	12 (1.4)	
7	597 (9.1)	508 (8.9)	89 (10.1)	
8	417 (6.3)	392 (6.9)	25 (2.8)	
9	479 (7.3)	413 (7.3)	66 (7.5)	
10	553 (8.4)	481 (8.4)	72 (8.2)	
11	942 (14.3)	790 (13.9)	152 (17.3)	
Waitlisted time (including inactive status), median (IQR), d	36 (9–189)	35 (9–189)	40 (8–183)	0.92
New allocation, *n* (%)				< 0.001
1	574 (8.7)	488 (8.6)	86 (9.8)	
2	3002 (45.6)	2579 (45.3)	423 (48.1)	
3	1341 (20.4)	1169 (20.5)	172 (19.6)	
4	1329 (20.2)	1189 (20.9)	140 (15.9)	
5	46 (0.7)	0 (0.0)	46 (5.2)	
6	287 (4.4)	274 (4.8)	12 (1.5)	
Posttransplant survival at 1-yr, %	91.3	92.6	83.3	< 0.001

BiVAD, biventricular assist device; BNP, B-type natriuretic peptide; BUN, blood urea nitrogen; CVP, central venous pressure; ECMO, extracorporeal membrane oxygenation; eGFR, estimated glomerular filtration rate; IABP, intraaortic balloon pump; IQR, interquartile range; LVAD, left ventricular assist device; NT-proBNP= N terminal pro BNP; RVAD, left ventricular assist device; TAH, total artificial heart; UNOS, United Network of Organ Sharing; VAD, ventricular assist device.

Data are presented as number (%), median (interquartile range) as appropriate.

a *P*-value applies to the comparison of 2 outcomes groups.

In Figure 2, we illustrate the “importance” scores for various features used in the final RF model. It is evident that the most important predictors include the eGFR, eGFR reported > 1 month prior, eGFR ratio (eGFR at listing/eGFR at transplant), blood urea nitrogen levels at listing, and both dialysis status and dialysis required > 1 month prior. The boxplots represent the distribution of the feature importance scores for these 15 predictive variables, with longer boxes and whiskers indicating a wider variation in the importance scores across the model iterations.

In Supplementary Figure S1, we show a precision/recall curve for the study cohort. This curve is a key tool in evaluating the performance of the prediction model, especially in scenarios with imbalanced datasets. It illustrates the trade-off between precision (the proportion of true positive results among all positive predictions, PPV) and recall (the proportion of true positive results detected among all actual positives, sensitivity) across different thresholds. This curve helps in understanding the balance between the precision and recall in the model's performance. Despite the low incidence of the adverse outcome (13%), the area under the curve for the precision/recall curve remained relatively high at 0.670.

**Figure 3:**
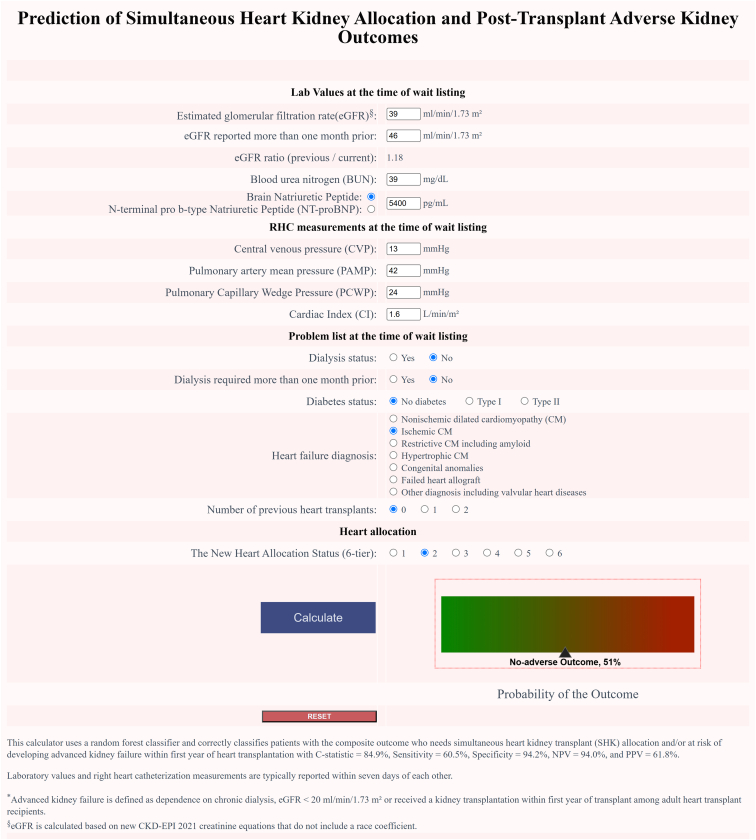
Web-portal for the patient level decision tool (https://shk-calc.web.app). RHC, right heart catheterization.

In Table 2, we show the diagnostic testing accuracy of various models. The models were evaluated in different settings, including a training set with 5-fold cross-validation (internal validation), 2 external validation cohorts. The sensitivity of these models ranged from 0.605 to 0.694 across different settings, while specificity remained high (∼ 0.934–0.955). The PPVs and NPVs varied slightly across different settings, with PPVs ranging from 0.604 to 0.680 and NPVs around 0.940 to 0.955. The c-statistics for these models ranged from 0.849 to 0.899, indicating reliable performance in the model’s ability distinguishing between classes. The F1 scores ranged from 0.611 to 0.680, indicating a good balance in the RF model. In Figure 3, we highlight the web-portal designed for the patient-level decision tool. This tool enables transplant providers to input patient-specific data and receive predictions based on the machine learning model.

**Table 2:** Diagnostic testing accuracy of the random forest model^a^ in the different study cohorts

Experiment	Setting	Dataset	Sensitivity	Specificity	PPV	NPV	C- statistic	P/R F1 Score
Training set (heart transplants performed between October 2018 and December 2020)	Original RF model with internal validation (5-fold cross validation)	*n* = 6579 True Pos = 880	0.605	0.942	0.618	0.940	0.849	0.611
Death censored training set cohort (excluding deaths within 1 yr posttransplant)	Using above original RF model	*n* = 5275 True Pos = 733	0.611	0.955	0.654	0.947	0.858	0.632
External validation cohort 1 (heart transplants performed between January 2021 and June 2021)	Using above original RF model	*n* = 1567 True Pos = 244	0.680	0.941	0.680	0.941	0.899	0.680
External validation cohort 2 (heart transplants performed between September 2023 and March 2024), postpolicy period (Medical Eligibility and Safety Net)	Using above original RF model	*n* = 1853 True Pos = 235	0.694	0.934	0.604	0.955	0.874	0.646

c-statistic, area under the receiver operating characteristic curve; NPV, negative predictive value; OPTN, Organ Procurement and Transplantation Network; P/R, precision/recall; Pos, positive cases; PPV, positive predictive value; RF, random forest; SHKT, simultaneous heart kidney transplant.

a During the model training phase, we selected the best performing model (as measured by area under the curve) from an exhaustive search over a range of 32 to 256 embedded decision trees (in increments of 32), a maximum tree depth ranging from 1 to 10 (increments of 1), a maximum number of features ranging from 1 to 5 when looking for the best split (increments of 1), a class weight for positive samples ranging from 3 to 10 (increments of 1), and using Gini index as the splitting criterion.

In Table 3, we show the examples of characteristics and outcomes in the external validation cohort (January to June 2021) and their corresponding predictions using a web-based patient-level decision tool based on the RF model (Figure 3), can be accessed at https://shk-calc.web.app.

**Table 3:** Characteristics and the outcomes of selected actual patients in the external validation cohort (January to June 2021) and their corresponding predictions using a web-based patient-level decision tool based on the random forest model

Random forrest model features and outcomes	Patient 1	Patient 2	Patient 3	Patient 4	Patient 5
eGFR (ml/min per 1.73 m^2^)	33	39	27	10^a^	37
eGFR (ml/min per 1.73 m^2^), > 1 mo earlier	32	35	32	10^a^	42
eGFR ratio	0.97	0.90	1.19	1.00	1.14
Dialysis need	No	No	No	Yes	No
Dialysis need, > 1 mo earlier	No	No	No	Yes	No
BUN (mg/dl)	24	39	43	54	20
Diabetes	Yes, type 2	No	Yes, type 2	Yes, type 1	Yes, type 2
Heart failure category	ICM	NICM	NICM	ICM	ICM
Number of previous HTs	0	0	0	0	0
BNP (pg/mL)	2708	1470	3821	11666	46
CVP (mmHg)	12	23	14	15	4
PAMP (mmHg)	29	37	30	46	14
PCWP (mmHg)	21	25	25	30	7
CI (L/min/m^2^)	1.8	2.3	2.2	3.6	2.0
New HT allocation status (6-tier)	2	1	3	3	2
Actual transplant performed	SHKT	HTA	HTA	HTA	HTA
Recommended transplant type based on the RF model prediction	SHKT	Indeterminate	SHKT	SHKT	HTA
The RF model binary outcome prediction and associated risk score	Positive, 65%	Equipoise, 50%	Positive, 80%	Positive, 93%	Negative, 59%
Adverse kidney outcomes within 1 yr posttransplant	No	No	Yes (On dialysis)	Yes (On dialysis)	No
Mortality within 1 yr posttransplant	No	No	No	Yes	No

BNP, B-type natriuretic peptide; BUN, blood urea nitrogen; CI, cardiac index; CVP, central venous pressure; eGFR, estimated glomerular filtration rate; HT, heart transplant; HTA, heart transplantation alone; ICM, ischemic cardiomyopathy; NICM, nonischemic cardiomyopathy; PAMP, pulmonary artery mean pressure; PCWP, pulmonary capillary wedge pressure; RF, random forest; SHKT, simultaneous heart kidney transplant.

The median time for the reported laboratory values (renal function testing and BNP) and right heart catheterization measurements were on the same day and within 5 days of each other, respectively.

a If a patient is on dialysis, it is assumed that eGFR = 10 ml/min per 1.73 m^2^.

## Discussion

Our RF model is tailored to assist in clinical decision-making during heart transplant wait listing, before determining between HT alone and SHKT. This RF model particularly integrates a range of factors such as kidney function and dialysis status at 2 data points, a heart failure biomarker, invasive hemodynamics, and the latest HT allocation tiers. The training model using a contemporary study cohort ensured its relevance and effectiveness in current medical practices. The diagnostic accuracy of our RF model, as evidenced by its performance across various cohorts, demonstrates remarkable metrics, including c-statistic, high specificity, and NPV. These metrics highlight the model's strong capability in accurately identifying patients less likely to benefit from SHKT and to develop adverse kidney outcomes post-transplant. The consistent and strong performance across both external validation cohorts underscore the model’s robustness and reliability. These results support its potential for broad clinical application as a supplementary tool at the time of listing.

Our model demonstrates good specificity, NPV, and discrimination, suggesting that it performs well in ruling out negative cases and maintaining reliability in certain predictive aspects. We acknowledge that the sensitivity (0.605–0.680) and PPV (0.618–0.680) are moderate; however, these metrics are influenced by the underlying outcome prevalence (13%) and the potential impact of unmeasured variables in the retrospective dataset. In cases where the positive incidence is low, the PPV can appear lower, even in well-calibrated models. When the RF model predicts an adverse binary outcome, caution should be exercised, and incorporating additional clinical data such as proteinuria, kidney imaging, and biopsy findings along with serial RF predictions may enhance decision-making. This is particularly important because of the short median wait time for HT (4–5 weeks) and the dynamic nature of various factors, despite previous prospective testing. The online tool is intended to supplement, not replace, clinical judgment in addressing the complexities of SHKT decision-making at the time of waitlisting. It may warrant multiple risk assessments using the predictive tool during transplant evaluation, particularly for patients with borderline GFR values.

Deciding whether to list patients with moderate kidney failure for SHKT is a complex process, particularly because of the implications of the new heart allocation policy. The difficulty arises from several considerations: choosing among HT alone, SHKT, the use of mechanical circulatory assist devices (such as left/right/biventricular assist devices, extracorporeal membrane oxygenation, total artificial heart, and intraaortic ballon pump) as a bridge to transplant or selecting recipients and appropriate donors. Furthermore, the SHKT procedure itself can negatively impact posttransplant kidney function. Challenges include adverse perioperative hemodynamics, such as heart graft dysfunction or hypotension requiring extensive use of vasopressors or inotropes, issues of volume overload, bleeding, or early postoperative complications and use of calcineurin inhibitors. In the context of SHKT, kidney graft is particularly susceptible to ischemic injury and has a higher risk of early graft failure from these complications, compared with heart graft. Our RF model, incorporating BNP levels, right heart catheterization measurements, and the new HT allocation status, may allow for a better assessment of the pretransplant risks of adverse renal outcomes associated with right ventricular dysfunction in patients on mechanical circulatory assist device.

We conducted additional experiments where we excluded patients who died in the first year following transplant. Our results, based on a death-censored cohort, demonstrated that the predictive model remains robust regardless of whether death is included. Although additional competing risk analyses could be conducted, it is unlikely that death as a competing risk would affect the model's performance.

On September 28, 2023, the OPTN approved new criteria for “Medical Eligibility and a Safety Net for SHKT and Simultaneous Lung-Kidney Transplantation”,^17^ closely mirroring the guidelines established in 2017 for simultaneous liver-kidney transplants.^18^ Under these criteria, a HT candidate qualifies for SHKT if they have AKI lasting ≥ 6 consecutive weeks (indicated by the need for dialysis or an eGFR < 25 ml/min per 1.73 m^2^), or CKD characterized by an eGFR < 60 ml/min per 1.73 m^2^ for ≥ 3 months, and an eGFR < 30 ml/min per 1.73 m^2^ at the time of joining the transplant waiting list or during the waiting period. It is anticipated that > 90% of SHKT listings will meet the CKD criteria, paralleling the experience with the simultaneous liver-kidney transplants policy and supported by recent unpublished United Network for Organ Sharing data for both SHKT and simultaneous lung-kidney transplantation (97.5%).^18^ In addition, the policy includes enhanced prioritization for HT recipients who develop severe, enduring kidney failure between 2 and 12 months posttransplant, as part of the Safety Net provision.

There may be questions about whether our RF model trained on older, center-specific SHKT allocation practices can accurately predict SHKT need under the current OPTN Medical Eligibility and Safety Net criteria. Model performance in the September 2023 to March 2024 postpolicy external validation cohort was comparable with the initial testing cohort, with slightly higher sensitivity (0.694 vs. 0.605), a modest decrease in specificity (0.934 vs. 0.942), lower PPV (0.604 vs. 0.618), improved NPV (0.955 vs. 0.940), and a slightly higher c-statistic (0.874 vs. 0.849). In this postpolicy cohort (*n* = 1853 HTs), 163 patients underwent SHKT. The observed composite outcome—SHKT recipients plus 72 patients with adverse posttransplant kidney outcomes—totaled 235 patients. In terms of aggregate estimation, the model predicted 269 patients would experience this outcome, an overestimation of 14.5% (269/235 = 1.145). When using a more refined benchmark (using actual post–Medical Eligibility and Safety Net data) that includes SHKT recipients and 86 safety net–eligible patients (*n* = 249) for the same period, the model’s overestimation decreases to ∼8% (269/249 = 1.08), reflecting closer alignment with postpolicy eligibility criteria.”

Although some retrospective cohort analyses suggest a survival advantage for SHKT when pretransplant eGFR is < 33 to 37 ml/min per 1.73 m^2^, a direct comparison of outcomes between SHKT and HT alone is fraught with challenges.^5^^,^^20^^,^^29^ These cohorts typically involve diverse patient populations, each with unique baseline characteristics and varying patterns of disease progression. Furthermore, substantial variations in eGFR are often observed, attributable to cardiorenal syndrome. Though SHKT qualifying requirement of a single permissive pretransplant eGFR < 30 ml/min per 1.73 m^2^ has the virtue of simplicity, a single eGFR measurement in time insufficiently predicts the severity and reversibility of kidney injury.^30^ In the external validation cohort, Table 3 demonstrates the utility of our RF algorithm in supporting decision-making for complex cases with low eGFR values. Patients 1, 2, and 5 had no adverse outcomes posttransplant, whereas patients 3 and 4 faced significant complications, including dialysis needs and mortality (for patient 4 only). The RF model's predictions corresponded well with actual outcomes, especially for those with higher risk scores where SHKT was recommended. The model's value stems from its training on data from patients not on dialysis who had adverse outcomes, with a median GFR of 30.3 ml/min/1.73 m^2^ and an interquartile range of 22.6 to 39.2 ml/min per 1.73 m^2^.

The allocation of deceased donor kidneys in a multiple organ transplant setting is a complex and highly debated issue because it often takes precedence over the allocation of kidneys to candidates requiring a kidney-alone transplant. Multiple organ transplants, particularly for simultaneous liver-kidney transplants and SHKTs, negatively affect candidates for kidney-alone transplant by reducing their access to high-quality kidneys. Approximately 30% of kidney-alone transplant candidates who would have received a kidney that was instead given to a multiple organ transplant recipient either died or were removed from the waiting list before receiving a transplant. These bypassed candidates faced a 55% higher risk of mortality.^12^ In addition, > 60% of SHKT recipients, despite being in the highest-acuity categories and receiving high-quality kidneys, experienced significant 90-day mortality and primary nonfunction rates. This suggests that the allocation, which is based on heart disease severity rather than kidney disease, is flawed. The policies governing SHKT require regular monitoring and revision to ensure equitable access to organs for kidney-alone transplant patients. Several proposals^8^ have been put forth to address the current system's shortcomings. The core goal of these proposals is to improve the utility of the allocation system, thereby creating a fairer process for all patients on the waiting list.

AI plays a pivotal role in predicting survival and significant complications in HT and mechanical circulatory assist devices.^31^, ^32^, ^33^, ^34^, ^35^, ^36^ The incorporation of AI in SHKT has the potential to transform the field, although current examples of such integration are few.^21^^,^^37^ Deploying AI in SHKT requires the availability of robust databases for model training and validation.^38^^,^^39^ Comprehensive datasets, encompassing clinical data, biomarkers, imaging, and histopathology are essential for fine-tuning AI models specific to SHKT allocation. It is crucial that AI models are not only effective but also transparent and understandable to health care providers, facilitating their widespread adoption and effective use.^22^

We acknowledge several limitations in our research. First, our retrospective study design relied on national registry data, which lacks several unmeasured variables that could have influenced our findings. These include detailed information on recipient kidney dysfunction (e.g., imaging, biopsy, and functional testing), regional or center-specific differences. We acknowledge that the COVID-19 pandemic may have influenced donor and recipient selection at transplant centers during the study period. Although the true impact at the center level is difficult to predict, an increase in the aggregate number of heart transplants and SHKT in the US between 2020 and 2024 suggests the pandemic's effect was likely modest (Figure 1). In addition, our model could not incorporate donor characteristics, because this information is unavailable at the time of waitlisting. Finally, the database lacks variables to differentiate between acute kidney disease and CKD or the duration of pretransplant dialysis in heart transplant recipients. These data limitations likely account for the low sensitivity of our predictive model.

In conclusion, the tool is intended to supplement, not replace, clinical judgment in addressing the complexities of SHKT decision-making at the time of waitlisting. It may warrant multiple risk assessments using the predictive tool during transplant evaluation, particularly for patients with borderline GFR values.

## Disclosure

All authors declared no competing interests.

## Supplementary Material

Supplementary File (PDF)

**Figure S1**. Precision /recall (P/R) curve for the study cohort.

**Table S1**. Pretransplant recipient characteristics selected by 3 domain experts (a heart transplant surgeon, a cardiologist, and a nephrologist) using the UNOS-STAR waitlist and transplant files.

**Table S2**. Characteristics of the heart transplant alone recipients who received transplant between October 18, 2018 and December 31, 2020 in the United States.

**Table S3**. Characteristics of the simultaneous heart kidney transplant (SHKT) recipients who received transplant between October 18, 2018 and December 31, 2020 in the United States.

**CONSORT flow diagram**.

**STROBE checklist**.
