# Supplementary material for: Predicting Simultaneous Heart Kidney Allocation and Posttransplant Adverse Kidney Outcomes
Source: Kidney Int Rep. 2025 Oct 15;11(1):152–63. doi: 10.1016/j.ekir.2025.10.005 (PMC12799570; doi:10.1016/j.ekir.2025.10.005)
Supplement: Supplementary File (PDF) [file mmc1.pdf]

Supplemental Table:

Table S1. Pre-transplant recipient characteristics selected by three domain experts (a heart transplant surgeon, a cardiologist and a nephrologist) using the UNOS-STAR wait list and transplant files.

1. Age at listing
2. Sex at listing
3. Ethnicity / Race at listing
4. History of diabetes at listing
5. Highest education level at listing
6. Previous heart transplant at listing
7. Primary insurance at listing
8. History of cigarette use at listing
9. Primary diagnosis at listing
10. Symptomatic cerebrovascular disease at listing
11. ABO blood type at listing
12. Panel reactive antibody at listing
13. United Network for Organ Sharing Region at listing
14. Serum creatinine (mg/dl) at listing and pre-transplant
15. Estimated glomerular filtration rate (eGFR) (ml/min/1.72 m^2) according to the Chronic Kidney Disease-Epidemiology Collaboration (CKD-EPI) equation (race neutral 2021 equation) at listing and pre-transplant
16. Blood urea nitrogen (BUN) mg/dl at listing
17. B-type natriuretic peptide (BNP)
18. Dialysis at listing and pre-transplant
19. Functional status (Karnofsky score, %) at listing and pre-transplant
20. Weight (kg) at listing and pre-transplant
21. Height (cm) at listing and pre-transplant
22. Body mass index (kg/m^2) at listing and pre- transplant
23. Cardiac output (L/min) at listing and pre-transplant
24. Previous cardiac surgery (non-heart transplant) at listing and pre-transplant
25. Number of previous heart transplants
26. Mechanical ventilation at listing and pre-transplant

27. Extra corporeal membrane oxygenation (ECMO) at listing and pre-transplant
28. Intra-aortic ballon pump (IABP) at listing and pre-transplant
29. Implantable cardioverter defibrillators (ICD) at listing and pre-transplant
30. Ventricular assist device (VAD) type at listing and pre-transplant
31. Central venous pressure (CVP) (mmHg), at listing and pre-transplant
32. Pulmonary capillary wedge pressure (mmHg), at listing and pre-transplant
33. Pulmonary artery systolic pressure (mmHg), at listing and pre-transplant
34. Pulmonary artery diastolic pressure (mmHg), at listing and pre-transplant
35. Pulmonary artery mean pressure (mmHg), at listing and pre-transplant
36. Cardiac index at listing and pre-transplant (calculated from cardiac output and body surface area)
37. Infection requiring IV antibiotics two weeks pre-transplantation
38. Patient location (Intensive care unit (ICU), non-ICU, not hospitalized) pre-transplant
39. Ebstein Bar virus (EBV) serostatus pre-transplant
40. Cytomegalo virus (CMV) serostatus pre-transplant
41. Hepatitis C virus (HCV) status pre-transplant
42. Human immunodeficiency virus (HIV) status pre-transplant
43. Bilirubin level pre-transplant
44. New heart allocation status (1-6 tiers)

Table S2. Characteristics the heart transplant alone recipients who received transplant between 10/18/2018 and 12/31/2020 in the United States.

	**Heart Transplant Alone Study Cohort**	**Heart Transplant Alone Without Adverse Renal Outcome Within One-Year Post-Transplant**	**Heart Transplant Alone with Adverse Renal Outcome Within One-Year Post-Transplant**	***P*-value***
N (%)	6,039 (100)	5,699 (94.4)	340 (5.6)	
Age, median (IQR) years	57 (45,63)	56 (45,63)	58 (50,65)	<0.001
Sex (male), n (%)	4363 (72.3)	4145 (72.7)	218 (64.1)	0.001
Race, n (%)				<0.001
White	3845 (63.7)	3653 (64.1)	192 (56.5)	
Black	1370 (22.7)	1258 (22.1)	112 (32.9)	
Hispanic	551 (9.1)	531 (9.3)	20 (5.9)	
Asian	210 (3.5)	196 (3.4)	14 (4.1)	
Other	63 (1.0)	61 (1.1)	2 (0.6)	
Recipient height (cm), mean (SD)	173.8 (10.1)	173.9 (10.1)	172.9 (10.7)	0.08
Recipient weight (kg), mean (SD)	84.3 (18.4)	84.2 (18.4)	86.1 (17.8)	0.07
Body surface area (m^2), mean (SD)	2.0 (0.2)	2.0 (0.2)	2.0 (0.2)	0.40
Body mass index (kg/m^2)	27.8 (5.0)	27.7 (5.0)	28.7 (5.0)	<0.001
History of diabetes, n (%)				0.001
No	4408 (73.0)	4189 (73.5)	219 (64.4)	
Type I	53 (0.9)	49 (0.9)	4 (1.2)	
Type II	1578 (26.1)	1461 (25.6)	117 (34.4)	

Etiology of heart failure, n (%)				0.08
Non-Ischemic	3402 (56.3)	3214 (56.4)	188 (55.3)	
Ischemic	1624 (26.9)	1540 (27.0)	84 (24.7)	
Restrictive	257 (4.3)	233 (4.1)	24 (7.1)	
Hypertrophic	204 (3.4)	197 (3.5)	7 (2.1)	
Congenital	338 (5.6)	315 (5.5)	23 (6.8)	
Failed heart transplant	144 (2.4)	133 (2.3)	11 (3.2)	
Others	70 (1.2)	67 (1.2)	3 (0.9)	
Previous heart transplant, n (%)				0.52
0	5881 (97.4)	5552 (97.4)	329 (96.8)	
1	150 (2.5)	139 (2.4)	11 (3.2)	
2	8 (0.1)	8 (0.1)	0 (0.0)	
Cardiac index (L/min/m^2), mean (SD)	2.2 (0.6)	2.2 (0.6)	2.2 (0.6)	0.23
Central venous pressure (mmHg), mean (SD)	9.2 (5.5)	9.2 (5.4)	10.6 (6.0)	<0.001
Pulmonary capillary wedge pressure (mmHg), mean (SD)	17.9 (8.3)	17.8 (8.3)	18.2 (8.3)	0.44
Pulmonary artery mean pressure (mmHg), mean (SD)	27.1 (9.2)	27.0 (9.2)	28.1 (9.4)	0.04
Mechanical ventilation requirement, n (%)	131 (2.2)	120 (2.1)	11 (3.2)	0.17
ECMO, n (%)	202 (3.3)	189 (3.3)	13 (3.8)	0.61

IABP, n (%)	940 (15.6)	884 (15.5)	56 (16.5)	0.64
VAD, n (%)				0.86
None	4398 (72.8)	4155 (72.9)	243 (71.5)	
LVAD alone	1536 (25.4)	1447 (25.4)	89 (26.2)	
RVAD	9 (0.2)	8 (0.1)	1 (0.3)	
TAH	18 (0.3)	17 (0.3)	1 (0.3)	
BiVAD	78 (1.3)	72 (1.3)	6 (1.8)	
B-type natriuretic peptide (BNP)				
BNP (pg/ml), mean (SD) n=2931	962.3 (1244.2)	961.6 (1242.3)	975.0 (1285.9)	0.90
NT Pro BNP (pg/ml), mean (SD) n=2321	4020.5 (5243.1)	3960.9 (5159.8)	4915.0 (6317.6)	0.03
BUN (mg/dl) at listing, mean (SD)	23.1 (11.2)	23.0 (11.2)	25.8 (12.4)	<0.001
eGFR ml/min/1.73 m^2 at listing	69.5 (24.1)	70.1 (24.1)	59.4 (22.4)	<0.001
eGFR ml/min/1.73 m^2 prior to transplant	69.7 (25.6)	70.7 (25.4)	54.3 (24.8)	<0.001
eGFR ratio (wait listing /prior to transplant)	1.0 (0.5)	1.1 (0.5)	1.0 (0.4)	<0.001
Dialysis at listing, n (%)	30 (0.5)	22 (0.4)	8 (2.4)	<0.001
Dialysis prior to transplant, n (%)	107 (1.8)	83 (1.5)	24 (7.1)	<0.001
eGFR ml/min/1.73 m^2 prior to transplant, if not on dialysis, median (IQR)	67.8 (52.3, 88.2)	68.7 (52.9, 88.9)	56.0 (41.5, 71.4)	<0.001
UNOS Region				0.01

1	351 (5.8)	328 (5.8)	23 (6.8)	
2	596 (9.9)	562 (9.9)	34 (10.0)	
3	693 (11.5)	663 (11.6)	30 (8.8)	
4	521 (8.6)	490 (8.6)	31 (9.1)	
5	953 (15.8)	901 (15.8)	52 (15.3)	
6	174 (2.9)	171 (3.0)	3 (0.9)	
7	545 (9.0)	508 (8.9)	37 (10.9)	
8	404 (6.7)	392 (6.9)	12 (3.5)	
9	440 (7.3)	413 (7.3)	27 (7.9)	
10	505 (8.4)	481 (8.4)	24 (7.1)	
11	857 (14.2)	790 (13.9)	67 (19.7)	
Waitlisted time (including inactive status), median (IQR), days	35 (9,189)	35 (9, 189)	25 (7,177.5)	0.89
New Allocation, n (%)				0.19
1	520 (8.6)	488 (8.6)	32 (9.4)	
2	2752 (45.6)	2579 (45.3)	173 (50.9)	
3	1234 (20.4)	1169 (20.5)	65 (19.1)	
4	1246 (20.6)	1189 (20.9)	57 (16.8)	
5	0 (0.0)	0 (0.0)	0 (0.0)	
6	287 (4.8)	274 (4.8)	13 (3.8)	
Post-transplant survival at one-year, %	91.7	92.6	76.8	<0.001

Abbreviations: CVP= central venous pressure; ECMO= extracorporeal membrane oxygenation; IABP= intra-aortic balloon pump; VAD= ventricular assist device; LVAD= left ventricular assist device; BiVAD= biventricular assist device; TAH= total artificial heart; BNP= B-Type natriuretic peptide; BUN= blood urea nitrogen; eGFR= estimated glomerular filtration rate; UNOS= United Network of Organ Sharing.

*p-value applies to the comparison of between two outcome groups

Data are presented as number (%), median (interquartile range) as appropriate. Laboratory values (renal function testing and BNP) and right heart catheterization measurements were typically reported on the same day and within five days of each other, respectively.

Table S3. Characteristics the simultaneous heart kidney (SHKT) transplant recipients who received transplant between 10/18/2018 and 12/31/2020 in the United States.

	**SHKT Study Cohort**	**SHKT without Adverse Renal Outcome Within One-Year Post-Transplant**	**SHKT with Adverse Renal Outcome Within One-Year Post-Transplant**	***P*-value***
N (%)	540 (100)	435 (80.6)	105 (19.4)	
Age, median (IQR) years	56 (48,63)	56 (48,63)	57 (47,63)	0.86
Sex (male), n (%)	404 (74.8)	322 (74.0)	82 (78.1)	0.39
Race, n (%)				0.02
White	237 (43.9)	198 (45.5)	39 (37.1)	
Black	208 (38.5)	159 (36.6)	49 (46.7)	
Hispanic	57 (10.6)	49 (11.3)	8 (7.6)	
Asian	25 (4.6)	22 (5.1)	3 (2.9)	
Other	13 (2.4)	7 (1.6)	6 (5.7)	
Recipient height (cm), mean (SD)	173.6 (10.0)	173.5 (10.3)	174.4 (8.8)	0.05
Recipient weight (kg), mean (SD)	82.6 (18.7)	82.2 (18.6)	84.2 (18.8)	0.33
Body surface area (m^2), mean (SD)	2.0 (0.2)	2.0 (0.2)	2.0 (0.2)	0.33
Body mass index (kg/m^2)	27.3 (5.1)	27.2 (5.0)	27.6 (5.4)	0.49
History of diabetes, n (%)				0.28
No	295 (54.6)	231 (53.1)	64 (61.0)	
Type I	11 (2.0)	10 (2.3)	1 (0.9)	
Type II	234 (43.3)	194 (44.6)	40 (38.1)	

Etiology of heart failure, n (%)				0.70
Non-Ischemic	258 (47.8)	200 (46.0)	58 (55.2)	
Ischemic	159 (29.4)	134 (30.8)	25 (23.8)	
Restrictive	24 (4.4)	20 (4.6)	4 (3.8)	
Hypertrophic	10 (1.9)	9 (2.1)	1 (1.0)	
Congenital	19 (3.5)	16 (3.7)	3 (2.9)	
Failed heart transplant	66 (12.2)	53 (12.2)	13 (12.4)	
Others	4 (0.7)	3 (0.7)	1 (1.0)	
Previous heart transplant, n (%)				0.93
0	474 (87.8)	383 (88.1)	91 (86.7)	
1	57 (10.6)	45 (10.3)	12 (11.4)	
2	9 (1.7)	7 (1.6)	2 (1.9)	
Cardiac index (L/min/m^2), mean (SD)	2.4 (0.7)	2.4 (0.7)	2.3 (0.6)	0.18
Central venous pressure (mmHg), mean (SD)	11.3 (6.3)	11.1 (6.2)	12.5 (6.8)	0.05
Pulmonary artery mean pressure (mmHg), mean (SD)	29.8 (8.6)	29.5 (8.5)	31.2 (8.7)	0.07
Pulmonary capillary wedge pressure (mmHg), mean (SD)	20.2 (8.2)	19.7 (8.0)	21.9 (8.6)	0.02
Mechanical ventilation requirement, n (%)	3 (0.6)	2 (0.5)	1 (1.0)	0.54
ECMO, n (%)	17 (3.2)	14 (3.2)	3 (2.9)	0.85

IABP, n (%)	77 (14.3)	59 (13.6)	18 (17.1)	0.35
VAD, n (%)				0.32
None	427 (79.1)	340 (78.2)	87 (82.9)	
LVAD alone	95 (17.6)	81 (18.6)	14 (13.3)	
RVAD	3 (0.6)	3 (0.7)	0 (0.0)	
TAH	4 (0.7)	2 (0.5)	2 (1.9)	
BiVAD	11 (2.0)	9 (2.1)	2 (1.9)	
B-Type natriuretic peptide (BNP)				
BNP (pg/ml), mean (SD) n=269	1752.8 (2627.7)	1841.3 (2834.2)	1312.4 (1052.2)	0.22
NT pro-BNP (pg/ml), mean (SD)n=177	11808.4 (12068.3)	11426.1 (12077.4)	13416.2 (12075.7)	0.39
BUN (mg/dl) at listing, mean (SD)	45.6 (21.5)	45.5 (21.4)	46.2 (21.8)	0.74
eGFR ml/min/1.73 m^2 at listing	24.9 (16.3)	25.2 (16.5)	23.7 (15.1)	0.40
eGFR ml/min/1.73 m^2 prior to transplant	22.6 (17.5)	22.1 (15.8)	24.6 (23.3)	0.20
eGFR ratio (wait listing /prior to transplant)	1.0 (0.5)	1.0 (0.5)	1.1 (0.6)	0.13
Dialysis at listing, n (%)	182 (33.8)	147 (33.9)	35 (33.3)	0.92
Dialysis prior to transplant, n (%)	251 (46.8)	200 (46.4)	51 (48.6)	0.69
eGFR ml/min/1.73 m^2 prior to transplant, if not on dialysis, median (IQR)	30.3 (22.6, 39.2)	30.2 (22.7, 38.3)	32.0 (19.3, 44.2)	0.03
UNOS Region				0.001

1	29 (5.4)	26 (6.0)	3 (2.9)	
2	27 (5.0)	19 (4.4)	8 (7.6)	
3	59 (10.9)	47 (10.8)	12 (11.4)	
4	55 (10.2)	49 (11.3)	6 (5.7)	
5	124 (23.0)	100 (23.0)	24 (22.9)	
6	9 (1.7)	6 (1.4)	3 (2.9)	
7	52 (9.6)	41 (9.4)	11 (10.5)	
8	13 (2.4)	8 (1.8)	5 (4.8)	
9	39 (7.2)	23 (5.3)	16 (15.2)	
10	48 (8.9)	47 (10.8)	1 (1.0)	
11	85 (15.7)	69 (15.9)	16 (15.2)	
Waitlisted time (including inactive status), median (IQR), days	47.5 (10,190)	50 (10, 193)	39 (10,161)	0.60
New Allocation, n (%)				0.28
1	54 (10.0)	45 (10.3)	9 (8.6)	
2	250 (46.3)	192 (44.1)	58 (55.2)	
3	107 (19.8)	91 (20.9)	16 (15.2)	
4	83 (15.4)	67 (15.4)	16 (15.2)	
5	46 (8.5)	40 (9.2)	6 (5.7)	
6	0 (0.0)	0 (0.0)	0 (0.0)	
Post-transplant survival at one-year, %	87.4	87.8	85.7	0.56

Abbreviations: ECMO= extracorporeal membrane oxygenation; IABP= intra-aortic balloon pump; VAD= ventricular assist device; LVAD= left ventricular assist device; BiVAD= biventricular assist device; TAH= total artificial heart; BNP= B-Type natriuretic peptide; NT pro-BNP= N terminal pro-B-type natriuretic peptide BUN= blood urea nitrogen; eGFR= estimated glomerular filtration rate; UNOS= United Network of Organ Sharing.

*p-value applies to the comparison between two outcome groups.

Data are presented as number (%), median (interquartile range) as appropriate. Laboratory values (renal function testing and BNP) and right heart catheterization measurements were typically reported on the same day and within five days of each other, respectively.

Supplemental Figures:

Figure S1. Precision /Recall (P/R) curve for the study cohort.

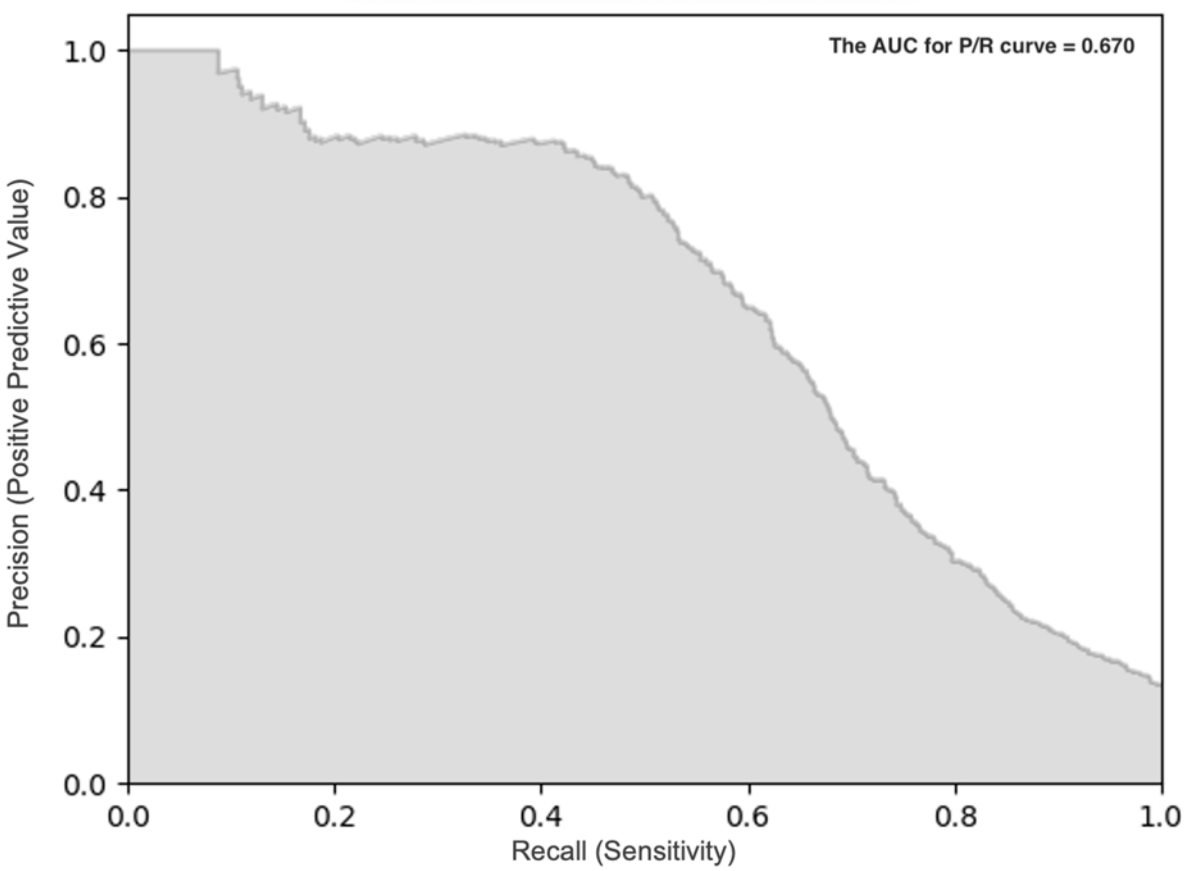

CONSORT Flow Diagram

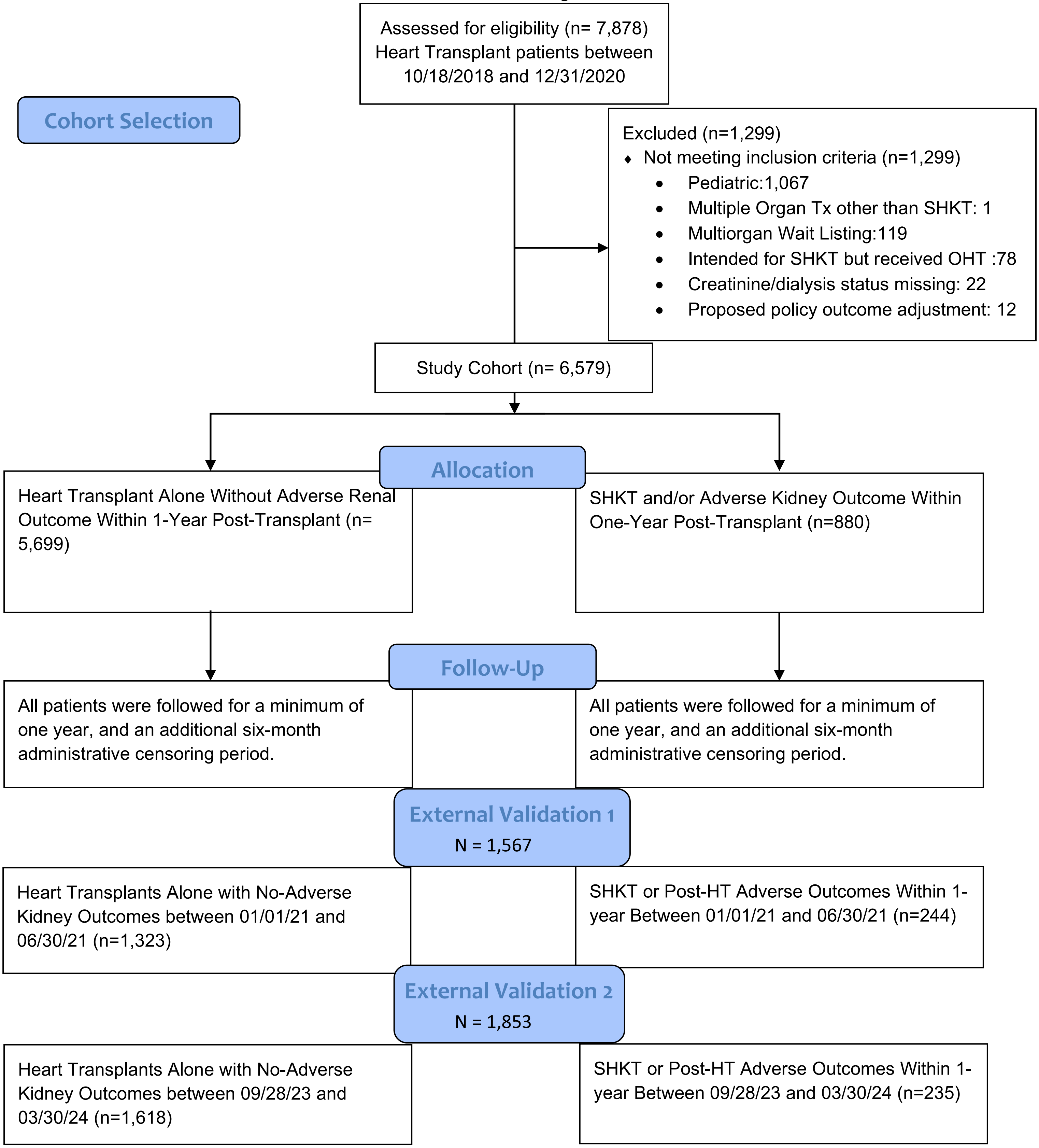

STROBE Statement—Checklist of items that should be included in reports of ***cohort studies***

	Item No	Recommendation	Page No
**Title and abstract**	1	(*a*) Indicate the study's design with a commonly used term in the title or the abstract<br>(*b*) Provide in the abstract an informative and balanced summary of what was done and what was found	1-4
**Introduction**			
Background/rationale	2	Explain the scientific background and rationale for the investigation being reported	5-6
Objectives	3	State specific objectives, including any prespecified hypotheses	6
**Methods**			
Study design	4	Present key elements of study design early in the paper	7
Setting	5	Describe the setting, locations, and relevant dates, including periods of recruitment, exposure, follow-up, and data collection	7
Participants	6	(*a*) Give the eligibility criteria, and the sources and methods of selection of participants. Describe methods of follow-up<br>(*b*) For matched studies, give matching criteria and number of exposed and unexposed	7-8
Variables	7	Clearly define all outcomes, exposures, predictors, potential confounders, and effect modifiers. Give diagnostic criteria, if applicable	7-8
Data sources/ measurement	8*	For each variable of interest, give sources of data and details of methods of assessment (measurement). Describe comparability of assessment methods if there is more than one group	7-9
Bias	9	Describe any efforts to address potential sources of bias	10
Study size	10	Explain how the study size was arrived at	7
Quantitative variables	11	Explain how quantitative variables were handled in the analyses. If applicable, describe which groupings were chosen and why	8-10
Statistical methods	12	(*a*) Describe all statistical methods, including those used to control for confounding<br>(*b*) Describe any methods used to examine subgroups and interactions<br>(*c*) Explain how missing data were addressed<br>(*d*) If applicable, explain how loss to follow-up was addressed<br>(*e*) Describe any sensitivity analyses	9-10
**Results**			
Participants	13*	(a) Report numbers of individuals at each stage of study—eg numbers potentially eligible, examined for eligibility, confirmed eligible, included in the study, completing follow-up, and analysed<br>(b) Give reasons for non-participation at each stage<br>(c) Consider use of a flow diagram	11-12
Descriptive data	14*	(a) Give characteristics of study participants (eg demographic, clinical, social) and information on exposures and potential confounders<br>(b) Indicate number of participants with missing data for each variable of interest<br>(c) Summarise follow-up time (eg, average and total amount)	11-12
Outcome data	15*	Report numbers of outcome events or summary measures over time	11-12

Main results	16	(*a*) Give unadjusted estimates and, if applicable, confounder-adjusted estimates and their precision (eg, 95% confidence interval). Make clear which confounders were adjusted for and why they were included<br>(*b*) Report category boundaries when continuous variables were categorized<br>(*c*) If relevant, consider translating estimates of relative risk into absolute risk for a meaningful time period	11-12
Other analyses	17	Report other analyses done—eg analyses of subgroups and interactions, and sensitivity analyses	12
**Discussion**			
Key results	18	Summarise key results with reference to study objectives	13-17
Limitations	19	Discuss limitations of the study, taking into account sources of potential bias or imprecision. Discuss both direction and magnitude of any potential bias	18
Interpretation	20	Give a cautious overall interpretation of results considering objectives, limitations, multiplicity of analyses, results from similar studies, and other relevant evidence	13-18
Generalisability	21	Discuss the generalisability (external validity) of the study results	17
**Other information**			
Funding	22	Give the source of funding and the role of the funders for the present study and, if applicable, for the original study on which the present article is based	19

*Give information separately for exposed and unexposed groups.

Note: An Explanation and Elaboration article discusses each checklist item and gives methodological background and published examples of transparent reporting. The STROBE checklist is best used in conjunction with this article (freely available on the Web sites of PLoS Medicine at http://www.plosmedicine.org/, Annals of Internal Medicine at http://www.annals.org/, and Epidemiology at http://www.epidem.com/). Information on the STROBE Initiative is available at http://www.strobe-statement.org.
